# Design, Synthesis, and Characterization of Tracers and Development of a Fluorescence Polarization Immunoassay for Rapid Screening of 4,4′-Dinitrocarbanilide in Chicken Muscle

**DOI:** 10.3390/foods10081822

**Published:** 2021-08-06

**Authors:** Qidi Zhang, Ming Zou, Wanyu Wang, Jinyan Li, Xiao Liang

**Affiliations:** 1 College of Veterinary Medicine, Qingdao Agricultural University, No. 700 Changcheng Road, Qingdao 266109, China; zqdcau@qau.edu.cn (Q.Z.); mzou@qau.edu.cn (M.Z.); 20192113675@stu.qau.edu.cn (W.W.); 13730914323@163.com (J.L.); 2 Basic Medical College, Qingdao University, No. 308 Ningxia Road, Qingdao 266071, China

**Keywords:** DNC residue, FPIA, tracers, chicken muscle

## Abstract

The compound, 4,4′-dinitrocarbanilide (DNC), is the marker residue of concern in edible tissues of broilers fed with diets containing anticoccidial nicarbazin (NIC). In this study, 25 fluorescein-labeled DNC derivatives (tracers) are synthesized and characterized to develop a rapid fluorescence polarization immunoassay (FPIA) for the detection of DNC in chickens using DNC monoclonal antibodies (mAbs). The effect of the tracer structure on the sensitivity of the FPIA is investigated. Our results show that after optimization, the half maximal inhibitory concentrations (IC_50_) and limit of detection (LOD) of the FPIA in the buffer are 28.3 and 5.7 ng mL^−1^, respectively. No significant cross-reactivity (CR < 0.89%) with 15 DNC analogues is observed. The developed FPIA is validated for DNC detection in spiked chicken homogenates, and recoveries ranged from 74.2 to 85.8%, with coefficients of variation <8.6%. Moreover, the total time needed for the detection procedure of the FPIA, including sample pretreatment, is <40 min, which has not been achieved in any other immunoassays for DNC from literature. Our results demonstrate that the FPIA developed here is a simple, sensitive, specific, and reproducible screening method for DNC residues in chickens.

## 1. Introduction

Coccidiosis refers to the disease caused by protozoans of the genus *Eimeria* resulting in a wide range of injuries in the intestinal tracts of poultry [1,2]. Intestinal invasion by these protozoans disrupts feeding, digestive processes, and nutrient absorption and results in dehydration, blood loss, and increased susceptibility to other etiological agents. Together, these effects can result in significant economic losses in the poultry industry, as well as considerable cause distress to the animals [3].

Numerous types of coccidiostats have been developed, and nicarbazin (NIC) was the first such agent found to give satisfactory control of coccidiosis in chicken production facilities and has been in general use for this purpose since the 1960s. The most common form of NIC is an equimolar mixture of 4,4′-dinitrocarbanilide (DNC) and 2-hydroxy-4, 6-dimethyl pyrimidine (HDP) (Figure S1). Despite emerging drug resistance in protozoan parasite populations, NIC has maintained its effectiveness against all species of *Eimeria* [4]. However, NIC use as a coccidiostats is limited to the initial growth phases of chickens, since it causes adverse heat stress effects when administered to older birds and often results in their mortality. Moreover, long term human exposure to low levels of NIC can result in chronic toxicity [5]. In addition, NIC administered via feed in chickens results in the persistence of the DNC residues in edible tissues [6]. Hence, DNC is the marker residue used for the detection of NIC in edible chicken tissues. The maximum residue limits (MRL) of DNC in food matrices has been established by Food and Agriculture Organization (FAO), as well as by New Zealand for chicken muscle tissues at 200 μg kg^−1^ [7]. The MRL has been set at 200 μg kg^−1^ in China [8]. Japan has established an MRL of DNC at 20 µg Kg^−1^ for aquatic products.

The current analytic methods for the determination of DNC in food matrices utilize high performance liquid chromatography (HPLC) and HPLC-tandem mass spectrometry (MS/MS) [9,10,11,12,13]. These methods are accurate and sensitive, but require well-equipped laboratories, high capital expenditures, highly trained personnel, and generally involve time consuming sample preparation steps. Therefore, a rapid and efficient alternative detection method for screening large numbers of samples would streamline DNC screening efforts.

Immunoassay techniques are effective and economical alternatives to instrumental methods for DNC. The enzyme-linked immunosorbent assay (ELISA) is the most frequent choice [1,14,15], but it is a heterogeneous solid phase method and the time required to complete the assay is often >2 h, because of the need to separate the unbound probe in solution before the bound probe can be quantified [1]. In contrast, fluorescence polarization immunoassay (FPIA) is a competitive homogeneous assay that is based on differences in fluorescence polarization (FP) of the fluorescently-labeled analyte in the antibody bound and non-bound fractions (Figure S2). These reactions can reach equilibrium in minutes or even seconds, and no separation or washing steps are required, which makes it ideal for high-throughput screening of large numbers of samples [16,17]. These advantages of the FPIA have resulted in their widespread use in high-throughput screening of chemical contaminants, such as veterinary drugs [16,17,18,19], mycotoxins, pesticides, and other environmental contaminants in foods, feed, and environmental samples [20,21,22]. However, the application of FPIA to the detection of DNC has not been reported.

The top priorities in developing high-throughput screening methods are to simplify the assay and shorten analysis time, while maximizing the sensitivity of the methods [19]. With this aim, we developed a novel FPIA method for the screening of DNC in the present work. We investigated the effects of different tracers and physicochemical factors on the performance of the FPIA. The optimized immunoassay was compared with other published detection methods, and the newly developed method provided a simple, rapid, reproducible, and highly sensitive detection of DNC. Analysis of DNC in chicken samples required <40 min to complete using the FPIA.

## 2. Materials and Methods

### 2.1. Reagents and Equipments

NIC, 4-Nitroaniline, 2-Nitroaniline, 3-Nitroaniline, N-(4-Nitrophenyl) propionamide, H-Val-Pna HCl, L-Argininep-Nitroanilide Dihydrochloride, 4-Nitrophenethylamine hydrochloride, N-Methyl-4-nitrophenethylamine hydrochloride, H-Ala-Pna HCl, N,N-Dimethyl-4-Nitroaniline, H-Glu-Pna, Halofuginone, Toltrazuril, 1, 3-Diphenylguanidine, Ronidazole, and dinitolmide were obtained from Sigma-Aldrich (St. Louis, MO, USA). N, N-Dimethylformamide (DMF), 1-ethyl-3-(3-dimethylaminopropy) carbodiimide (EDC), N-hydroxysuccinimide (NHS), fluorescein isothiocyanate, ethylenediamine, butanediamine, hexamethylenediamine, 5-Aminofluorescein (5-AF), and 6-Aminofluorescein (6-AF) were obtained from Aladdin (Shanghai, China). Nonbinding-surface black microplates were purchased from Corning Life Sciences (New York, NY, USA). Normal solvents and salts were of analytical reagent grade and were supplied by Beijing Reagent Corporation (Beijing, China). The haptens DNC-1, DNC-2, DNC-3, DNC-4, and DNC-5 and four mAbs (4E1, 4B8, 2A12, and 3B4) were acquired from China Agricultural University [23]. Borate buffer (0.05 M, pH 8.0) was used as the working buffer for all FPIA experiments. The standard solution of DNC (2 mg mL^−1^) was prepared by dissolving 4 mg of the DNC standard in 2 mL of dimethyl sulfoxide, and stored at −20 °C until use.

A SpectraMax M5 microplate reader from Molecular Devices (Downingtown, PA, USA) was used to measure FP. Black microplates (96-well) with a non-binding surface for FPIA were purchased from Corning Life Sciences (New York, NY, USA). Water was purified using a Milli-Q system from Millipore Inc. (Bedford, MA, USA). A ultraviolet analyzer was obtained from Tianjin Huike Instrument Equipment Co., Ltd. (Tianjin, China).

### 2.2. Preparation of FITC-DNC Tracers

**Figure 1:**
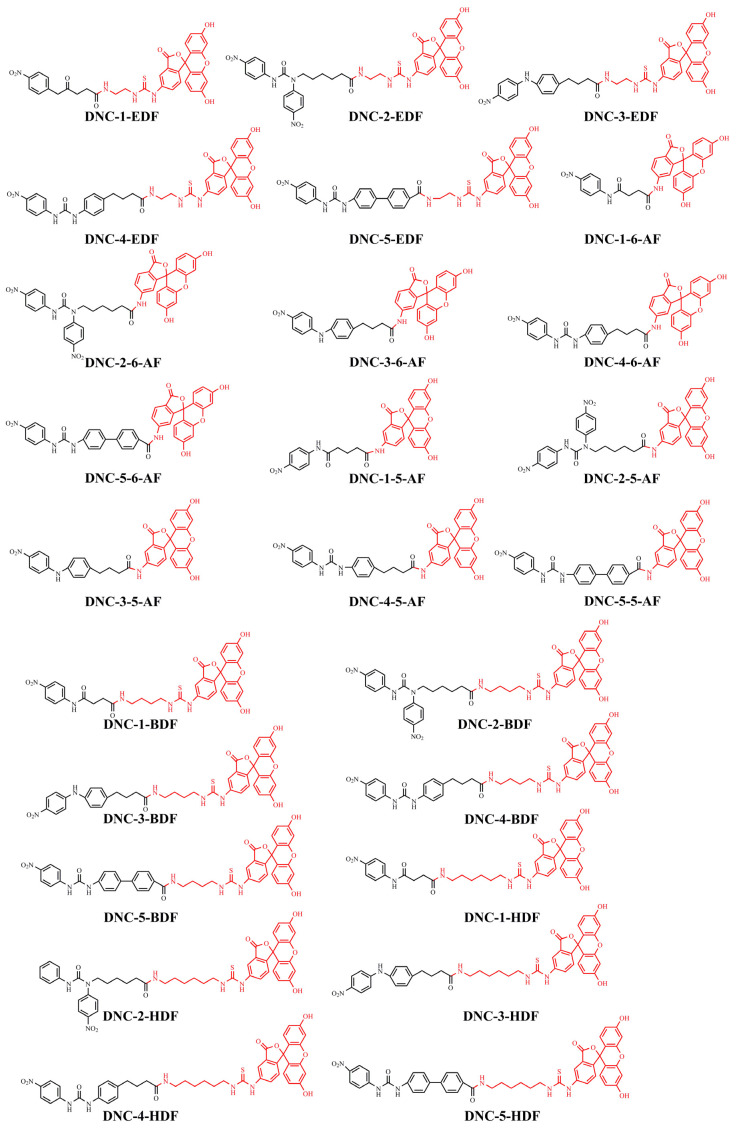
Structures of 25 different tracers of DNC.

Thiocarbamoyl ethylenediamine fluorescein (EDF), thiocarbamoyl butane diamine fluorescein (BDF), and thiocarbamoyl hexane fluorescein (HDF) were described previously [16]. Synthesis of the tracers used in the current study has been described elsewhere [24]. Briefly, 10 mg of hapten (DNC-1, DNC-2, DNC-3, DNC-4, or DNC-5) dissolved in 500 μL DMF was mixed with 30 mg NHS and 40 mg of EDC. After stirring at room temperature overnight, the reaction mixture was centrifuged to remove the precipitate at 5000× *g* for 10 min. Then 10 mg EDF was added to the supernatant and stirred overnight at room temperature. An aliquot (50 μL) of the mixture was purified using thin layer chromatography (TLC) with methanol/ trichloromethane (1:6, *v/v*) as eluent. The major TLC bands possessed a retardation factor (R_f_) of 0.70, and they were collected and stored in methanol at 4 °C in the dark (Figure S3). In total, 25 tracers (Figure 1) for DNC were generated, and those bound to the specific mAbs were chosen for further study (see below).

### 2.3. FPIA Procedure

The tracer solutions were diluted with borate buffer to working concentrations with FP values 10 times that of the borate buffer background. The FPIA approach was described, as follows—70 μL of each tracer solution and 70 μL DNC standard solution or borate buffer were added to 70 μL antibody in a microplate well. After incubation for 20 min in the dark at room temperature, the FP value of the mixture was measured at λ_ex_ 485 nm and λ_em_ 530 nm (emission cutoff = 515 nm), respectively.

The antibody-tracer pair used in the final assay was the DNC-4-BDF and mAb 3B4 combination. The concentration of DNC-4-BDF was 200 RFU, and the dilution of mAb 3B4 was 1/300. The incubation time was 20 min at room temperature. The physicochemical conditions of the reaction buffer were pH 8 and NaCl concentration at 0 mM, respectively.

### 2.4. Curve Fitting and Statistical Analysis

A sigmoidal curve was used to fit FPIA data via OriginPro 8.0 (Origin Lab, Northampton, MA, USA) for construction of the standard curves. A four-parameter logistic equation was used to fit the immunoassay data as follows:

Y = (A − D)/[1 + (X⁄C)*^B^*] + D (1)

where A and D represent the maximum and minimum values, respectively; B is the slope factor; C is the concentration corresponding to 50% specific binding (IC_50_), and X is the calibration concentration [25]. The limit of detection (LOD) was the concentration of the standard causing 10% inhibition of tracer binding (IC_10_), and the working range corresponded to concentrations of the standard from IC_20_ to IC_80_ on the calibration curve. The IC_50_ and LOD values were used to evaluate the properties of the FPIA.

The specificity of the immunoassays was evaluated by determining the cross-reactivity (CR) with DNC analogs under the optimized conditions. CR was calculated with the following equation:

CR (%) = (IC_50_ of DNC/IC_50_ of DNC analog) × 100 (2)

### 2.5. FPIA Development and Optimization

The standard test procedure is outlined in Section 2.3 (see above). In brief, respective antibody tracer pairs were selected using the detection window according to the equation δmP = mP_bound_ − mP_free_ where IC_50_ values for the FPIA and the IC_50_/δmP ratio were used as the main parameters for the selection of optimum antibody tracer pairs. Tracer solutions were diluted with borate buffer to working concentrations that possessed fluorescence intensity (FI) values 10× above background. Specifically, diluted antibody and tracer that possessed a suitable δmP (≥90 mP) was evaluated, and the IC_50_ and IC_50_/δmP values were determined and adjusted to minimize the IC_50_ and IC_50_/δmP values and were calculated according to standard curves for DNC (see above). The FPIA was optimized by determining the influences of antibody dilution, reaction time, pH, salt concentration, and organic solvent levels on assay characteristics. The δmP and IC_50_ values were used as the primary criteria to evaluate FPIA performance.

### 2.6. Chicken Sample Analysis for FPIA

Samples of chicken muscle (2 g) were homogenized and then extracted with 2 mL methanol at room temperature. The mixtures were vortexed vigorously for 10 min and centrifuged at 10,000 rpm for 10 min at 4 °C. The supernatant was diluted 5-fold with assay buffer prior to analysis. For recovery experiments, blank chicken matrix samples obtained from WDWK Biotech (Beijing, China) were fortified with NIC at 50, 100, and 150 μg kg^−1^, and 5 replicates were analyzed at each concentration using the optimized FPIA.

## 3. Results and Discussion

### 3.1. Synthesis and Characterization of FITC-DNC Tracers

**Figure 2:**
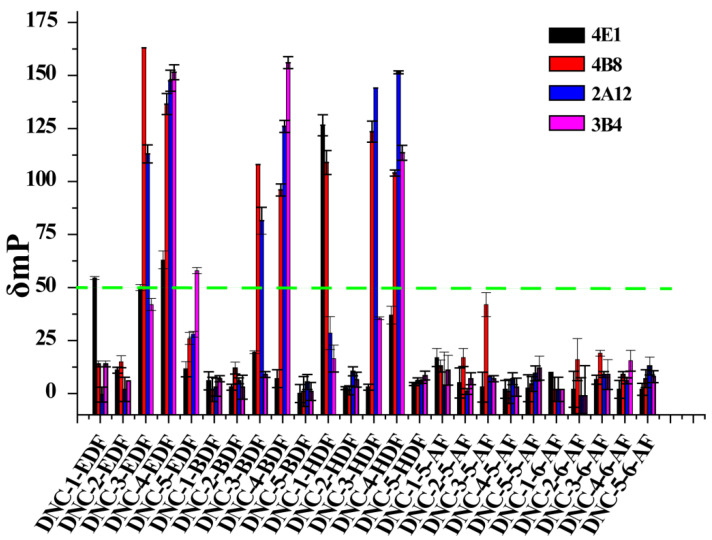
Characterization of the fluorescein-labeled DNC conjugates.

**Figure 3:**
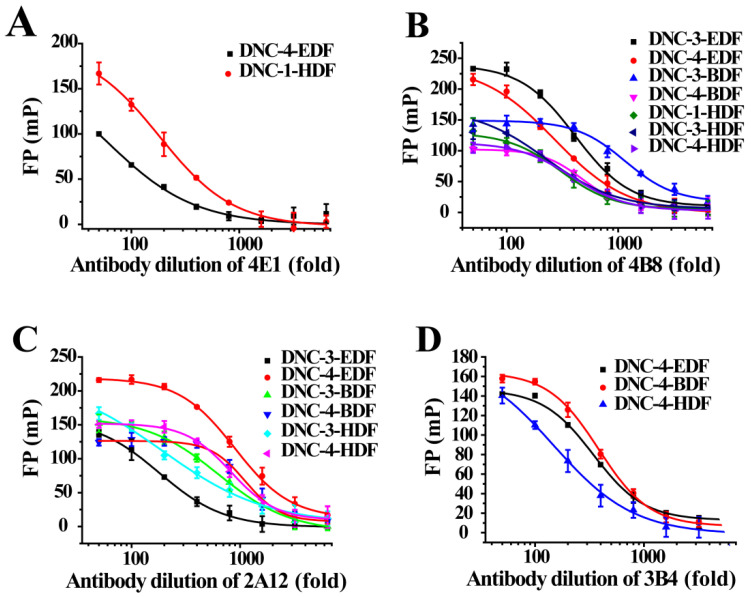
Antibody dilution curves of tracers. (**A**) 4E1 with DNC-4-EDF and DNC-1-HDF. (**B**) 4B8 with DNC-3-EDF, DNC-4-EDF, DNC-3-BDF, DNC-4-BDF, DNC-1-HDF, DNC-3-HDF, and DNC-4-HDF. (**C**) 2A12 with DNC-3-EDF, DNC-4-EDF, DNC-3-BDF, DNC-4-BDF, DNC-3-HDF, and DNC-4-HDF. (**D**) 3B4 with DNC-4-EDF, DNC-4-BDF, and DNC-4-HDF.

The assay tracers EDF, BDF, and HDF possessed molecular ion peaks (*m*/*z*) of 450, 478.03, and 506.06, respectively, demonstrating that the fluorescein conjugates were synthesized successfully (Figure S4). These tracers play key roles in the FPIA, since both the hapten type and the bridge length between the hapten and the FITC can markedly influence antibody recognition. Therefore, we examined five structurally different haptens and five fluorescein molecules containing different carbon bridges and synthesized 25 tracers to evaluate FPIA performance. The high molecular polarities of the haptens and fluorescein resulted in high R_f_ values on TLC when methanol/trichloromethane (1:6, *v/v*) was used as the developing solvent. After the separation and purification of the tracers with TLC, they were characterized using FPIA. There were nine tracers that displayed obvious binding to the specific mAbs (δmP > 50 mP) and possessed significant immunochemical activity indicative of successful tracer conjugation (Figure 2). Within this group, DNC-1-EDF and DNC-5-EDF possessed the lowest δmP values (54 and 57 mP, respectively), indicating that they were not suitable for developing a sensitive FPIA. The remaining seven tracers (DNC-3-EDF, DNC-4-EDF, DNC-3-BDF, DNC-4-BDF, DNC-1-HDF, DNC-3-HDF, and DNC-4-HDF) and four MAbs (4E1, 4B8, 2A12 and 3B4) were used to construct antibody dilution curves (Figure 3A–D). Satisfactory binding (δmP = 94~236 mP) was observed for all these tracers when combained with each of the 4 mAbs. Thus, these seven tracers were selected for further study.

### 3.2. FPIA Development and Optimization

#### 3.2.1. Selection of Antibody—Tracer Pairs

**Figure 4:**
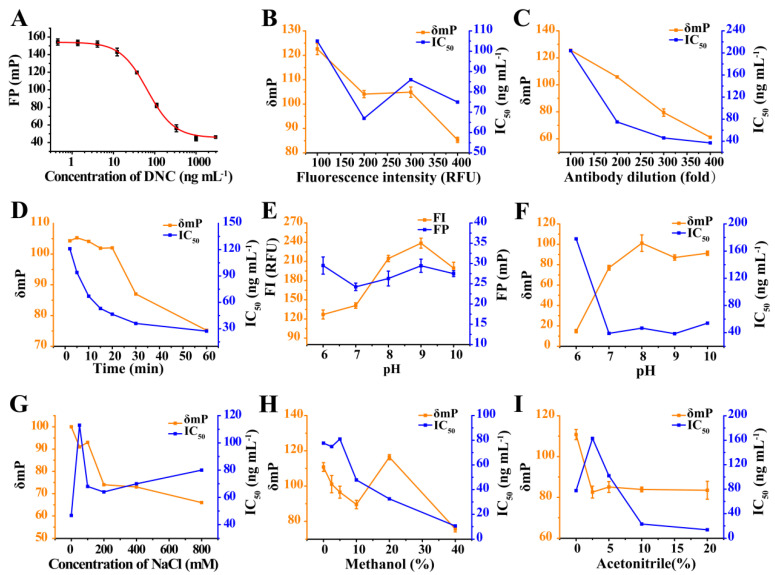
(**A**) Standard curves for DNC using DNC-4-BDF and mAb 3B4 combination. (**B**) Optimization of fluorescence intensity. (**C**) Optimization of antibody dilution. (**D**) Optimization of incubation time. (**E**) The effects of pH on FI and FP values. (**F**) Optimization of pH. (**G**) Optimization of NaCl concentration. (**H**) Effects of methanol concentration on assay performance. (**I**) Effects of acetonitrile concentration on assay performance.

The combination of tracer and antibody has significant impacts on the sensitivity and specificity of an FPIA [26]. We evaluated the best tracer and antibody pair for use in the assay by constructing DNC standard curves. The optimum of mAb dilution was obtained from antibody dilution curves, and a δmP of 100 mP was set as the target detection range. In particular, the DNC-4-BDF and mAb 3B4 combination provided the lowest IC_50_ (66 ng mL^−1^) and IC_50_/δmP ratio (0.61) along with a broad detection window (δmP = 107 mP) (Figure 4A). Moreover, the Z′ factor of 0.89 represented a good separation for the distributions and indicated a robust FPIA. This combination was, therefore, selected to develop an FPIA for the detection of DNC (Table 1).

The use of heterologous tracers for FPIA significantly enhances its sensitivity [16,27], and we found similar results in our study. For example, mAb 4B8 was prepared using DNC-3-KLH as immunogen and was paired with the seven tracers [23]. The IC_50_ values (ng mL^−1^) for the FPIA with these structurally heterologous tracers were DNC-4-EDF (263), DNC-4-BDF (3370), DNC-1-HDF (91), DNC-4-HDF (4020). These values were generally lower than those for the FPIA generated with homologous tracers, such as DNC-3-EDF (26680), DNC-3-BDF (11360), and DNC-3-HDF (9960) (Figure S5 and Table 1). When the remaining three mAbs (i.e., 2A12, 3B4, and 4E1) were examined in a similar way using DNC-4-BSA, DNC-5-BSA, and DNC-1-BSA as immunogens and then paired with the seven tracers, similar results were obtained because heterologous tracers could be more easily replaced by competitors (Table 1).

The effects of the length of the linker chain between a given hapten and fluorescein on FPIA sensitivity were also investigated in this study. The primary differences were the linker structure or length, as well as the orientation of the attached fluorophore. The IC_50_ values (ng mL^−1^) for the FPIA increased when mAb 4B8 was paired with DNC-4-EDF (263), DNC-4-BDF (3370), and DNC-4-HDF (4020). The lowest IC_50_ was obtained with DNC-4-BDF when mAb 3B4 or 2A12 were paired with DNC-4-based tracers. The DNC-3-HDF tracer possessed the longest bridge and displayed the lowest IC_50_ when combined with mAb 4B8 or 2A12 (Table 1). Previous studies have indicated that sensitivity was optimal with long linkers between a hapten and fluorescein [19,28,29]. In contrast, FPIA based on short bridge tracers resulted in greater assay sensitivity [30,31]. Thus, both the structural features of the tracer hapten itself and the structure and length of the bridge between a hapten and fluorescein label markedly influence the recognition of the tracer by antibody, and it is necessary to select the optimal combination empirically.

#### 3.2.2. Optimization of Tracer and mAb Concentrations

A competitive FPIA was used to optimize antibody and tracer concentrations. For instance, a low tracer concentration would yield higher sensitivity, but would also reduce the precision of the FP signal. In our assays, the FI values for DNC-4-BDF at levels of 5, 10, 15, and 20-fold greater than background (FI, 20 relative fluorescence units (RFU)) were examined. The optimal working concentration was set at an FI value of 200 RFU that was 10-fold higher than background as the lowest IC_50_ value, and an appropriate δmP value (104 mP) was observed (Figure 4B). The working concentration of mAb 3B4 was optimized at a fixed amount of tracer according to the standard curves for DNC. Both the IC_50_ and δmP values increased as mAb 3B4 dilution increased, and the lowest IC_50_ value was observed with a dilution of 1/300. This δmP value (<80 mP) was too low for FPIA, and therefore, a 1/200 for mAb 3B4 was used because it retained a lower IC_50_ value, and an acceptable δmP value of 105 mP (Figure 4C).

#### 3.2.3. Optimization of Incubation Time

The incubation time for the assay must be chosen until equilibrium is established in the competition between an analyte and tracer [20]. We examined incubation times ranging from 2 to 60 min. It was found that the IC_50_ values decreased as incubation time increased from 2 min to 20 min and then plateaued at >20 min. The δmP value was almost constant with times <20 min and then decreased significantly at >20 min (Figure 4D). These results revealed that equilibrium was achieved at 20 min after the mixing of antibody and tracer; thus, 20 min was selected as the optimum incubation time.

#### 3.2.4. Optimization of Physicochemical Conditions

The effects of pH, ionic strength, and organic solvent tolerance were also assessed for the FPIA by comparing IC_50_ and δmP values under various reaction conditions. The dyes used in this assay were pH-sensitive materials, and their FI values increased in the pH range of 6 to 8 and then decreased at pH >8, although the FP values were not affected significantly by pH (Figure 4E,F). These results indicated that pH 6 was not suitable for the assay system, due to insufficient binding (δmP = 14). This implied that the developed FPIA could not tolerate acidic conditions. The IC_50_ values changed little at pH values ranging from 7 to 10, and the assay performed optimally at pH 8 with high sensitivity and maximal δmP (>100 mP). NaCl concentrations between 0 to 800 mM and negative effects on the IC_50_ and δmP were observed for NaCl concentrations from 0 to 800 mM (Figure 4G). High ionic strength most likely resulted in the disruption of antibody-antigen interactions. Therefore, NaCl was not included in the assay buffer. Methanol and acetonitrile added to the test system led to IC_50_ and δmP decreased in direct proportion to their concentrations. The presence of 20% methanol resulted in a significant increase of δmP (116 mP), and this solvent was generally better tolerated than acetonitrile. Acetonitrile concentrations exceeding 20% (final concentration) were not tolerated, although 20% acetonitrile was still tolerated (Figure 4H,I). If needed, the assay could be used at methanol concentrations of up to 20%.

### 3.3. Sensitivity and Cross-Reactivity of FPIA

**Figure 5:**
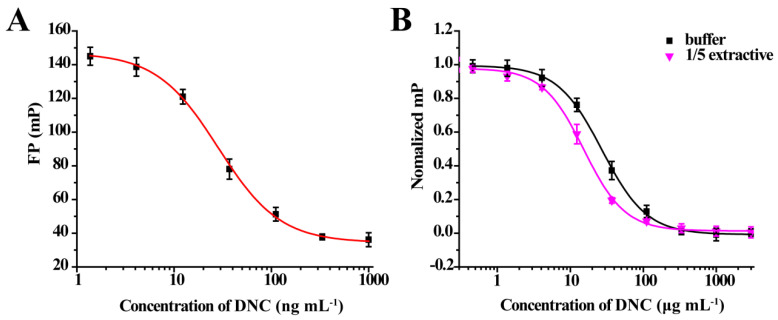
(**A**) Standard curves for NIC using the FPIA in assay buffer under optimized conditions. (**B**) Comparison of FPIA curves obtained from standards prepared in assay buffer, and 5-fold diluted chicken extracts.

Under the optimal conditions, the sensitivity and specificity of the FPIA were calculated using DNC and 15 DNC analogs. The IC_50_ value, the LOD defined as IC_10_ from the standard curve and linear range (IC_20_~IC_80_) were 28.39, 5.70, 10.31~78.17 ng mL^−1^, respectively (Figure 5A). The CR values for the developed FPIA were <0.89% for all 15 NIC analogs (Table 2). These results indicated that this FPIA was highly sensitive and specific for DNC.

### 3.4. Chicken Sample Analysis for FPIA

To further evaluate the utility of the FPIA, we determined recoveries of spiked tissue matrices. Calibration curves prepared in buffer, and diluted chicken extract were superimposed with normalization of the FP values indicating that the matrix interference was eliminated using 5-fold dilutions in methanol (Figure 5B). The mean recovery values for DNC at 50, 100, and 150 µg kg^−1^ ranged from 74.23 to 85.80% with CVs <8.64%. The LOD was 24.21 µg kg^−1^ and was sensitive enough to meet the detection requirements of MRL for DNC in chicken tissues set by the EU, the USA, and China. The working range of the FPIA was 31.15 to 188.35 μg kg^−1^ (Table 3). The sensitivity of the FPIA was lower than the ELISA developed using the same mAb (3B4) [23], but the FPIA, a homogeneous method, requires a much shorter time (less than 40 min) for the detection of DNC in chicken muscle. This is an essential characteristic needed for a rapid screening method. Compared with the required time of other immunoassays for DNC in animal-derived food or feeds, including ELISA (>2 h) [15], surface plasmon resonance (SPR) biosensor screening (>49 min) [32], time-resolved fluoroimmunoassays (TR-FIA) (>2.5 h) [33], and flow cytometry-based immunoassay (>2 h) [34], the shortest time was required for the new developed FPIA for DNC in chicken muscle.

## 4. Conclusions

In summary, an FPIA for DNC was established with favorable sensitivity, specificity, cost, time, and reliability for the first time. The sensitivity of the developed FPIA was significantly improved by optimizing the selection of tracers, tracer-antibody pairs, and physical and chemical reaction conditions. Furthermore, the reliability and robustness of the assay were successfully demonstrated for analysis of DNC in chicken muscle matrices. In addition, the sample pretreatment was simple for the developed FPIA. The total analysis time, including sample pretreatment, was less than 40 min, which has not yet been achieved in other immunoassays for DNC residues.

## Figures and Tables

**Table 1:** Analytical parameters of the standard curves were obtained using four anti-NIC mAbs with seven DNC tracers.

Tracers	mAbs	Dilution Fold	IC_50_ (ng mL^−1^)	δmP	IC_50_/δmP	Z′
DNC-3-EDF	2A12	100	1890	94	20.11	0.93
	4B8	400	26680	83.15	320.87	0.90
DNC-4-EDF	2A12	1800	5160	91.58	56.34	0.88
	4B8	500	263	96	2.74	0.87
	3B4	200	223	82.1	2.72	0.82
	4E1	50	7240	99.92	72.46	0.98
DNC-3-BDF	2A12	100	1520	98.72	15.39	0.92
	4B8	200	11360	71.45	158.99	0.94
DNC-4-BDF	2A12	400	1814	82.07	22.10	0.87
	4B8	100	3370	99	34.04	0.94
	3B4	200	66	107.76	0.61	0.89
DNC-1-HDF	4B8	200	91	89.28	1.02	0.84
	4E1	200	11600	75.65	153.33	0.81
DNC-3-HDF	2A12	200	290	133.07	2.18	0.91
	4B8	200	9960	68.85	144.66	0.87
DNC-4-HDF	2A12	700	4130	104.6	39.48	0.97
	4B8	200	4020	117.15	34.31	0.96
	3B4	100	2623	132.55	19.78	0.93

**Table 2:** IC_50_ values and Cross-reactivity of DNC and 15 structurally related analogs for the FPIA.

Analogues	Structure	IC_50_ (ng mL^−1^)	CR (%)
DNC	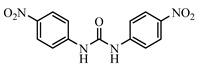	28.39	100
2-Nitroaniline	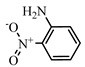	>30,000	<0.1
3-Nitroaniline	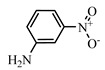	>30,000	<0.1
N-(4-Nitrophenyl) propionamide	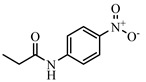	>30,000	<0.1
H-Val-Pna HCl	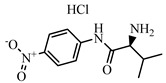	>30,000	<0.1
L-Arginine P-Nitroanilide Dihydrochl Oride	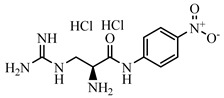	>30,000	<0.1
4-Nitrophenethylamine hydrochloride	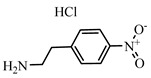	>30,000	<0.01
N-Methyl-4-nitrophenethylamine hydrochloride	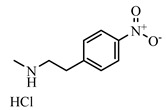	>30,000	<0.1
H-Ala-Pna HCl	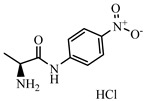	>30,000	<0.1
N, N-Dimethyl-4-Nitroaniline	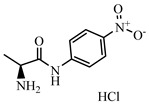	26,435	0.11
H-Glu-Pna	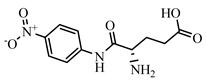	>30,000	<0.1
Halofuginone	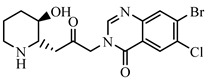	>30,000	<0.1
Toltrazuril	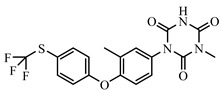	3190	0.89
1,3-Diphenylguanidine	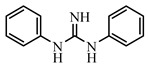	>30,000	<0.1
Ronidazole	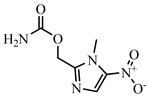	>30,000	<0.1
Dinitolmide	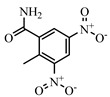	>30,000	<0.1

**Table 3:** Recovery studies from chicken muscle matrices using FPIA.

Sample	Spiked (μg kg^−1^)	Intra-Assay (n = 5)		Inter-Assay (n = 5)	
		Recovery (%)	CV (%)	Recovery (%)	CV (%)
Chicken	50	84.41	4.39	80.45	4.81
	100	82.6	5.12	85.80	4.00
	150	74.23	8.64	76.95	6.41

## Data Availability

Not applicable.

## Supplementary Materials

The following are available online at https://www.mdpi.com/article/10.3390/foods10081822/s1, Figure S1. The chemical structures of DNC and HDP, Figure S2. Schematic illustrations of the FPIA, Figure S3. The results of TLC for 25 tracers. (The red clip represents the target band that binds to the antibody), Figure S4. (A) Mass spectrum of EDF with molecular ion peak [M + 2H]^2+^ (*m*/*z*) of 450.00; (B) Mass spectrum of BDF with molecular ion peak [M + 2H]^2+^ (*m*/*z*) of 478.03; (C) Mass spectrum of HDF with molecular ion peak [M + 2H]^2+^ (*m*/*z*) of 506.06, Figure S5. Comparison of the effect of homologous and heterologous tracers on sensitivity.
