# Supplementary material for: Design, Synthesis, and Characterization of Tracers and Development of a Fluorescence Polarization Immunoassay for Rapid Screening of 4,4′-Dinitrocarbanilide in Chicken Muscle
Source: Foods. 2021 Aug 6;10(8):1822. doi: 10.3390/foods10081822 (PMC8392466; doi:10.3390/foods10081822)
Supplement: Supplementary file 1 [file foods-10-01822-s001.zip › foods-1310204-supplemantary.pdf]

Supporting Information for *Foods*

Design, synthesis and characterization of tracers and development of a fluorescence polarization immunoassay for rapid screening of 4, 4′ -dinitrocarbanilide in chicken muscle

Authors: Qidi Zhang [1, †], Ming Zou [1, †], Wanyu Wang [1], Jinyan Li [1], Xiao Liang [1, 2, *]

aInstitution and address:

1 College of Veterinary Medicine, Qingdao Agricultural University, No. 700 Changcheng Road, Qingdao 266109, People's Republic of China

bInstitution and address:

2 Basic Medical College, Qingdao University, No. 308 Ningxia road, Qingdao 266071, People's Republic of China

Correspondence: Xiao Liang Ph.D. College of Veterinary Medicine, Qingdao Agricultural University, No. 700 Changcheng Road, Qingdao 266109, People's Republic of China. Tel: +8613964867357; E-mail: liangxiao4000@163.com

†Co-author: Qidi Zhang and Ming Zou contributed equally to this work.

Contents

Figure S1. The chemical structures of DNC and HDP.

Figure S2. Schematic illustrations of the FPIA .

Figure S3. The results of TLC for 25 tracers. (The red clip represents the target band that binds to the antibody).

Figure S4. (A) Mass spectrum of EDF with molecular ion peak $[M+2H]^{2+}$ (m/z) of 450.00; (B) Mass spectrum of BDF with molecular ion peak $[M+2H]^{2+}$ (m/z) of 478.03; (C) Mass spectrum of HDF with molecular ion peak $[M+2H]^{2+}$ (m/z) of 506.06.

Figure S5. Comparison of the effect of homologous and heterologous tracers on sensitivity.

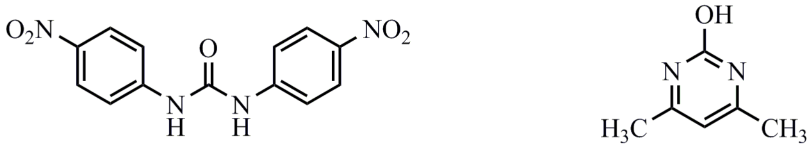

4, 4'- dinitro carbanilide
(DNC)

2-hydroxy- 4, 6- dimethyl pyrimidine
(HDP)

Figure S1. The chemical structures of DNC and HDP.

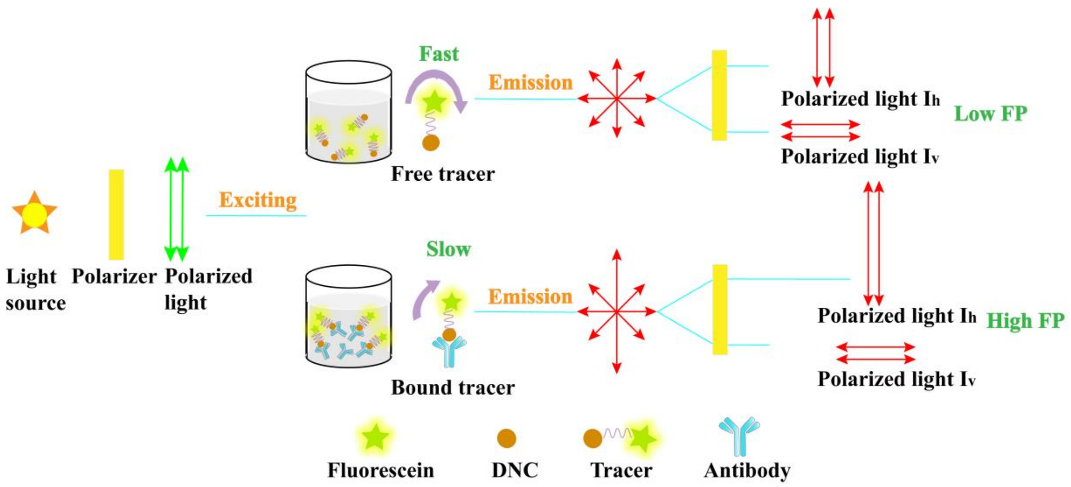

Figure S2. Schematic illustrations of the FPIA .

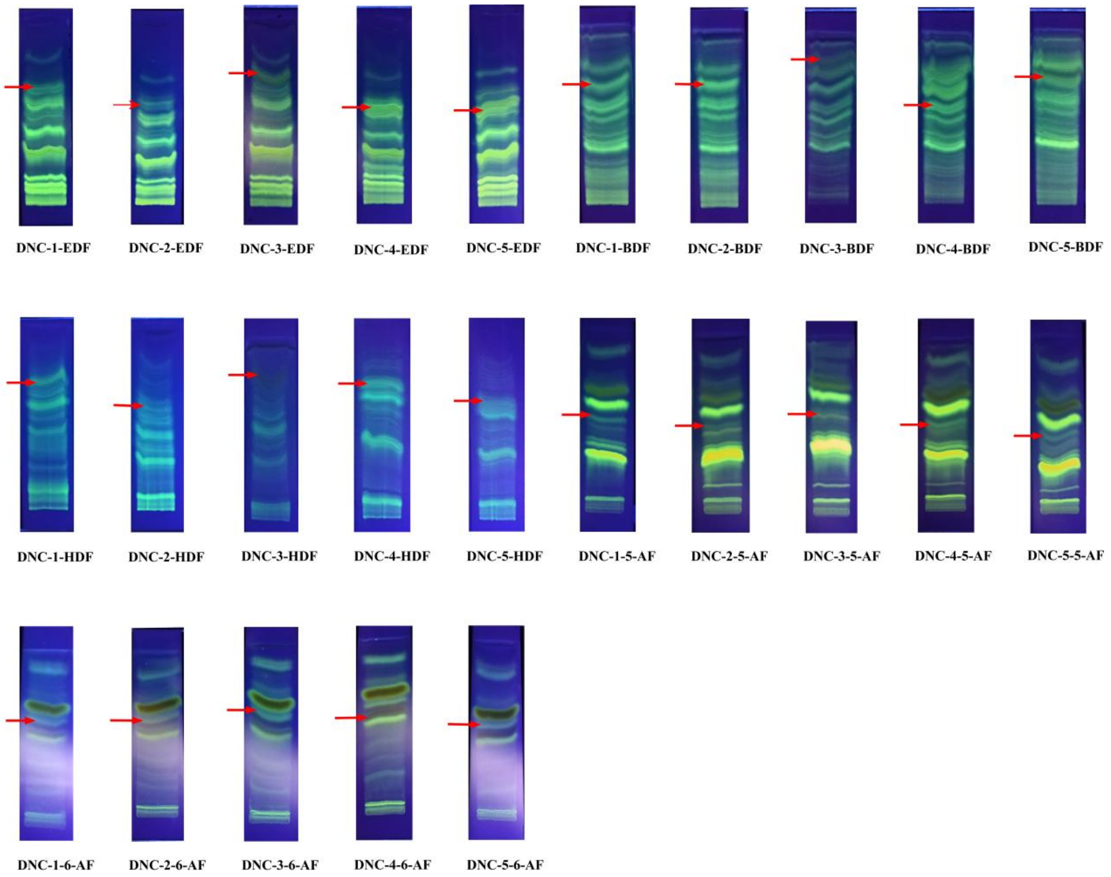

Figure S3. The results of TLC for 25 tracers. (The red clip represents the target band that binds to the antibody).

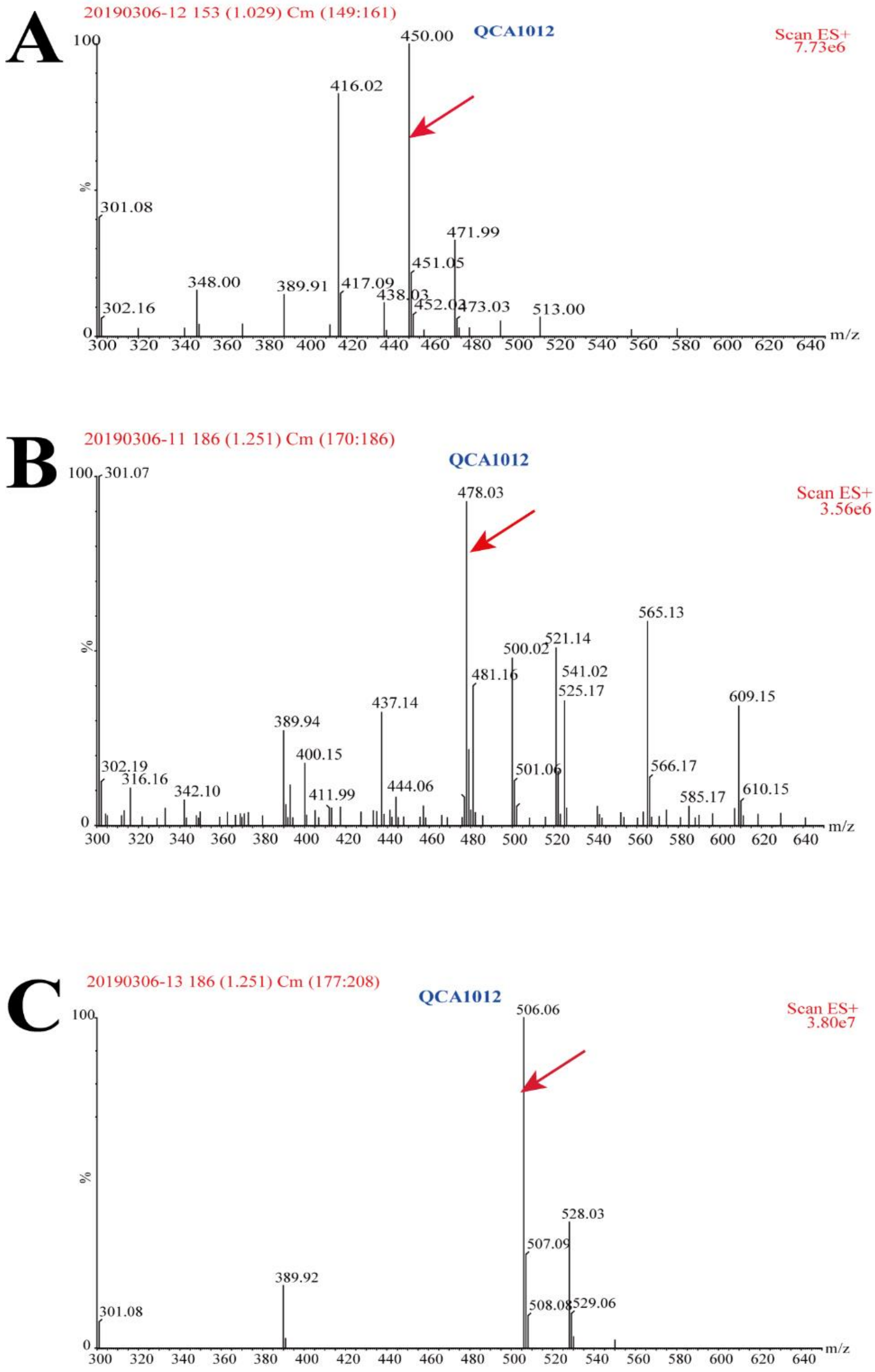

Figure S4 (A) Mass spectrum of EDF with molecular ion peak $[M+2H]^{2+}$ (m/z) of 450.00; (B) Mass spectrum of BDF with molecular ion peak $[M+2H]^{2+}$ (m/z) of 478.03; (C) Mass spectrum of HDF with molecular ion peak $[M+2H]^{2+}$ (m/z) of 506.06.

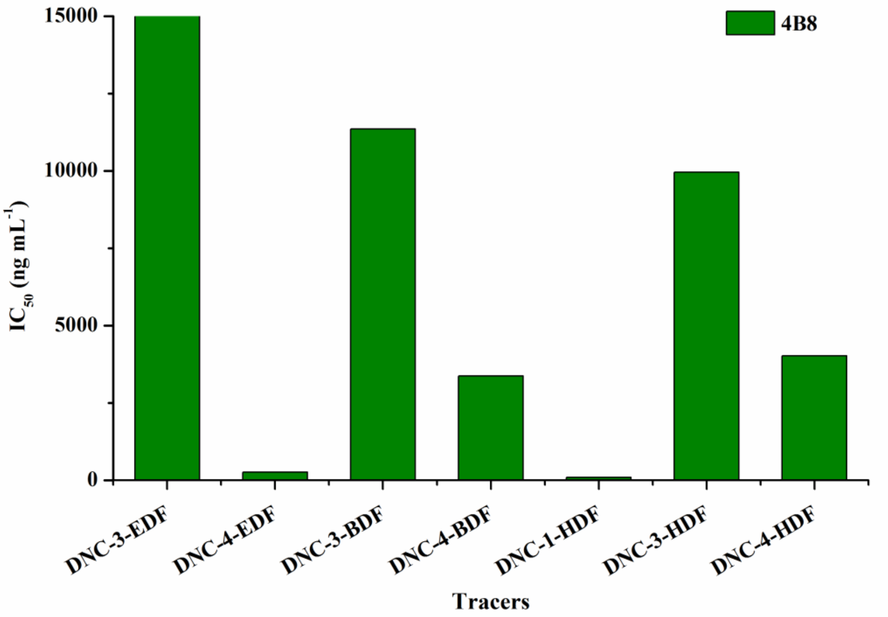

Figure S5. Comparison of the effect of homologous and heterologous tracers on sensitivity.
